# Single‐Cell and Bulk RNA Sequencing Highlights Intra‐Tumoral Heterogeneity and Malignant Progression Mechanisms in Prostate Cancer

**DOI:** 10.1111/jcmm.70806

**Published:** 2025-08-26

**Authors:** Junchao Wu, Ziqi Chen, Wentian Wu, Jiaxuan Qin, Rongfang Zhong, Jialin Meng, Yu Yin, Peng Guo, Song Fan

**Affiliations:** ^1^ Department of Urology, The First Affiliated Hospital of Anhui Medical University Hefei China; ^2^ Institute of Urology Anhui Medical University Hefei China; ^3^ Anhui Province Key Laboratory of Urological and Andrological Diseases Research and Medical Transformation Anhui Medical University Hefei China; ^4^ Department of Oncology The First Affiliated Hospital of Anhui Medical University Hefei China; ^5^ Department of Urology The University of Hong Kong‐Shenzhen Hospital Shenzhen China; ^6^ Department of Pathology The First Affiliated Hospital of Anhui Medical University Hefei China; ^7^ Department of Urology The Affiliated Jiangyin Hospital of Nantong University Wuxi China

**Keywords:** cell cycle, immunotherapy, intra‐tumour heterogeneity, prostate cancer, single‐cell RNA sequencing

## Abstract

Prostate cancer (PCa) is an extremely heterogeneous cancer and is highly prevalent in the older male population. Since intra‐tumour heterogeneity (ITH) commonly results in PCa chemotherapy resistance and recurrence, it is critical to explore its effects on tumour behaviour. Prognostic genes related to ITH were identified, and a signature was constructed using Cox regression analyses and multiple machine learning algorithms. Single‐cell RNA sequencing data extracted from PCa and CRPC samples were analysed via sub‐clustering, pseudotime, cell communication and drug sensitivity approaches to elucidate their function. The oncogenic potential of hub genes was confirmed by immunohistochemistry and cell proliferation assays. An 11‐gene signature underlying a prostate cancer meta‐program (PCMP) was generated by selecting an optimal combination of machine learning methods. Survival assays and multivariate Cox regression analyses conducted in multiple cohorts revealed the superior prognostic value of the PCMP signature. Functional enrichment analyses indicated that it dysregulates the cell cycle. Using trajectory and cell–cell communication analyses, we illustrated that PCMP genes exert oncogenic effects by enhancing the proliferation and oxidative phosphorylation of epithelial cells. Intra‐cellular assays also demonstrated that CENPA and CKS1B had promising malignant potential. In summary, our research not only establishes the association between the PCMP signature and reveals its malignant characteristics, but also deepens our understanding of the mechanisms underlying PCa progression and ITH. It holds promise for the development of targeted therapeutic interventions, thereby offering clinical benefits to patients.

**Abbreviations**
AUC: the average area under the curve
BCS: Beyondcell score
C‐index: Harrell's concordance index
CRPC: castration‐resistant prostate cancer
DKFZ: Deutsches Krebsforschungszentrum
EdU: 5‐ethynyl‐2′‐deoxyuridine
Enet: elastic network
GBM: generalised boosted regression modelling
GEO: gene expression omnibus
GO: gene ontology
IHC: immunohistochemistry
ITH: intra‐tumour heterogeneity
KEGG: Kyoto encyclopedia of genes and genomes
KM: Kaplan–Meier
MHC: major histocompatibility complex
MIF: macrophage migration inhibitory factor
OD: optical density
PCa: prostate cancer
PCMP: prostate cancer meta‐program
plsRcox: partial least squares regression for Cox
qPCR: quantitative polymerase chain reaction
RFS: recurrence free survival
RNA‐seq: RNA‐sequencing
RSF: random survival forest
scRNA‐seq: single‐cell RNA‐sequencing
siRNA: interfering RNA
ssGSEA: gene set enrichment analysis
SuperPC: supervised principal components
survival‐SVM: survival support vector machine
TCGA‐PRAD: Cancer Genome Atlas Prostate Adenocarcinoma
TME: tumour microenvironment
UMAP: uniform manifold approximation and projection
WB: Western Blot

## 1 Introduction

As the most common malignancy among older men, prostate cancer (PCa) imposes a substantial disease burden [1]. Based on a global cancer statistics report, there were almost 1.5 million new cases of PCa and 397,000 associated deaths worldwide in 2022 [2]. Currently available clinical treatment options for PCa include radical prostatectomy, androgen deprivation therapy and radiotherapy [3]. These treatment modalities are remarkably effective in the early stages. However, they may only succeed in slowing the progression of advanced PCa. The disease may ultimately recur and culminate in a more aggressive form known as castration‐resistant PCa (CRPC), which can progress to bone or distant organ metastases [4]. Moreover, conventional indicators, including prostate‐specific antigen levels and magnetic resonance imaging, exhibit low sensitivities [5]. Therefore, it is crucial to accurately predict PCa recurrence risk and explore therapeutic targets that can prevent the disease onset and progression.

Currently, immunotherapy has made significant progress in the treatment of solid tumours. Previous studies have demonstrated that intra‐tumour heterogeneity (ITH) tends to induce the formation of an immunosuppressive tumour microenvironment (TME), which leads to immunotherapy failure [6]. They have also confirmed the presence of different tumour subclones in the same patient, which contributes to the complexity of individual therapies [7, 8, 9]. As a malignant tumour with high inter‐tumour heterogeneity and ITH regarding histomorphology and molecular characteristics, PCa exists in multiple genomic and phenotypic forms [10, 11]. Based on previous studies, tumour heterogeneity and cellular plasticity have been identified as pivotal factors in actuating the malignant progression and the emergence of resistant phenotypes in PCa. Research has also demonstrated that the PCa cells within distinct statuses and anatomical regions exhibit unique characteristics [12, 13]. Through dissecting these cell populations and assessing their biological patterns, alongside their responses to various small‐molecular drugs, investigators have explored effective targeted inhibitors, such as Hepln‐13 and LAG‐3 inhibitors [14, 15], that can be applied to unique subclusters, thereby advancing the development of precision medicine. Nonetheless, the fundamental mechanisms governing this heterogeneity and plasticity remain largely unacknowledged; the evidence supporting the efficacy of targeted strategies against heterogeneous populations is currently not yet adequate. Therefore, a deeper investigation and rigorous validation of the intrinsic processes are imperative to fully understand and potentially overcome these challenges.

With the rapid development of genome and transcriptome sequencing technologies, the molecular landscape and marker genes for unique cancers have become increasingly clear. Routine transcriptome sequencing, also known as RNA‐sequencing (RNA‐seq), has emerged as a pivotal technique in genomic research, offering an exhaustive perspective on the transcriptional landscape of all cell types within a single sample. However, meticulous examination of distinct characteristics across diverse cell types may present a burden [16]. To address this limitation, single‐cell RNA‐seq (scRNA‐seq) can provide an impartial evaluation of heterogeneous cell populations and elucidate the discrete variabilities among individual cellular phenotypes within multifaceted cellular proportions [17]. Moreover, machine learning, a subset of artificial intelligence, is essential for discerning authentic and required objects from a multitude of variants [18, 19]. Consequently, the integration of single‐cell and bulk sequencing methodologies coupled with machine learning techniques can yield revolutionary insights into the early determination of intrinsic metastatic mechanisms at advanced stages.

In this study, we employed a comprehensive approach utilising 10 machine learning algorithms and their 101 combinations to build a PCa meta‐program (PCMP) model, which is designed to accurately predict the risk of recurrence and forward prognosis. Validated in multicentre PCa cohorts, the model exhibited superior predictive capacity. Furthermore, we investigated the potential mechanisms that drive heterogeneity and tumorigenesis. Moreover, for new insights into personalised treatment, we evaluated the sensitivity of different cell subsets to chemotherapy drugs, depicted using the Beyondcell R package [20]. The findings can provide valuable information for the precise diagnosis and treatment of PCa.

## 2 Materials and Methods

### 2.1 Dataset Acquisition and Preprocessing

Cancer Genome Atlas Prostate Adenocarcinoma (TCGA‐PRAD) bulk RNA‐seq data and relevant clinical information were downloaded from the Genomic Data Commons (TCGA). Samples with incomplete biochemical recurrence information were excluded to generate a training dataset of 490 patients with PRAD for further analysis. We transformed the transcripts per million data into a log2 format to achieve better comparability. To ensure the integrity of our analysis and the convenience for later verification, we searched the gene expression omnibus (GEO) and the Deutsches Krebsforschungszentrum (DKFZ) to acquire eligible datasets that meet the following criteria: (1) histopathologically confirmed diagnosis of PCa; (2) possession of high‐quality and available transcriptome data; (3) availability of essential clinical data, particularly RFS (recurrence free survival) and survival status. Samples failing to meet these criteria were excluded from the analysis. Then, the gene expression profiles were generated from six eligible datasets (GSE21032 [21], GSE70768, GSE70769 [22], GSE46602 [23], GSE116918 [24] and the DKFZ dataset).

The baseline characteristics are summarised in Table S1. The ‘sva’ package was employed to remove potential cross‐dataset batch effects along with the empirical Bayes framework. scRNA‐seq datasets (GSE193337 and GSE206962) containing four PCa and three CRPC samples were also extracted from the GEO database. Table S2 lists the detailed information pertaining to the single‐cell data.

### 2.2 Establishing a Prognostic Signature via Integrative 101 Machine Learning Approaches

Before model training, the raw transcriptomic data that was mentioned above underwent several preprocessing steps. This included the normalisation utilising TPM and the removal of lowly expressed genes to facilitate subsequent feature selection. After obtaining ITH meta‐programs [25], we employed univariate Cox regression analysis to filter out genes strongly correlated with relapse‐free survival in TCGA‐PRAD, GSE21032 and DKFZ, with a threshold of *p* < 0.05 and hazard ratios of the same direction for further analysis.

Considering overfitting, we utilised 10 algorithms, including random survival forest (RSF), elastic network (Enet), Lasso, Ridge, stepwise Cox, CoxBoost, partial least squares regression for Cox (plsRcox), supervised principal components (SuperPC), generalised boosted regression modelling (GBM), and survival support vector machine (survival‐SVM) to realise hybrid computing and construct a highly valuable prognostic model. Specifically, TCGA‐PRAD served as the training cohort for PCMP screening, whereas GSE70768, GSE70769, GSE21032, GSE46602 and DKFZ datasets were used as validation cohorts to check the accuracy of the prognostic model.

On the basis of the 10‐fold cross‐validation framework, 101 combinations of 10 algorithms were applied to narrow the range of prognostic genes [26], thereby mitigating overfitting. For each algorithm, we systematically explored a range of key parameters to identify the optimal configuration. For instance, we predominantly constructed the CoxBoost model, utilising the ‘optimCoxBoostPenalty’ function and determined the best number of boosting steps to conduct the machine learning process on the basis of 10‐fold cross‐validation. While in the case of SVM [27], we tested different kernels (linear, polynomial, RBF) and regularisation parameters (C values ranging from 0.1 to 100). Similarly, for random forests [28], we varied the number of trees (ranging from 100 to 1000) and the maximum depth of each tree. By determining the favourable parameters for each algorithm, we constructed and combined the best‐performing models. Other machine learning approaches, encompassing Enet, Lasso, Ridge, stepwise Cox, plsRcox, SuperPC and GBM, were all employed based on a 10‐fold cross‐validation framework; we investigated and selected the rational parameters for further analysis.

Subsequently, we calculated Harrell's concordance index (C‐index) and the average area under the curve (AUC) values for the 1‐, 3‐ and 5‐year RFS for each model. By comparing the C‐index and AUC in all cohorts, the optimal model with the highest value was selected. Through calculating the best risk score cut‐off value by the ‘survminer’ R package, patients were separated into high‐ and low‐risk groups.

### 2.3 Evaluation of the Clinical Significance of the PCMP Signature

Kaplan–Meier (KM) analysis was employed to demonstrate the prognostic potential of the PCMP signature. Univariate and multivariate Cox regression analyses were used to assess whether the PCMP served as an independent risk factor for patients with PCa. By calculating the C‐index and AUC for the risk score, along with other clinicopathological parameters, we validated the sensitivity and specificity of these indicators.

### 2.4 Gene Enrichment Analysis

The functional attributes of the PCMP signature were elucidated using gene ontology (GO) and Kyoto encyclopedia of genes and genomes (KEGG) enrichment analyses using the ‘clusterProfiler’ R package [29]. To discern perturbed signalling cascades, we downloaded hallmark gene sets from the Molecular Signatures Database (https://www.gsea‐msigdb.org/gsea/misgdb) and evaluated their abundance in TCGA samples utilising the single sample Gene Set Enrichment Analysis (ssGSEA) methodology [30]. The results were visualised utilising the ‘heatmap’ R package [31].

### 2.5 Comprehensive Immunological Profiling of Tumour Microenvironment

To investigate the correlation between TME and PCMPs, we conducted seven computational algorithms (CIBERSORT, MCPcounter, quantiseq, xCELL, IPS, TIMER and EPIC) to quantify the proportion of various immune cells using the ‘IOBR’ R package [32]. Alternatively, to assess their robustness, we calculated the immune infiltration landscape using ssGSEA and ESTIMATE algorithms [33, 34], thereby assessing consistency across the seven different methods. To compare patient responsiveness to immunotherapy, the two risk subgroups were compared for immune modulators, including co‐stimulators, co‐inhibitors, ligands, receptors, cell adhesion and antigen presentation [35].

### 2.6 Quality Control and Data Integration

Seurat (version 4.3.0) was used to process the scRNA‐seq data. To filter out high‐quality cells for each sample, cells with over 6000 or below 300 expressed genes, as well as those containing more than 20% mitochondrion‐derived unique molecular identifier counts, were removed. The top 2000 highly variable genes were identified using ‘FindVariableFeatures’. Next, the ‘Harmony’ package was used for performing sample batch correction. The ‘FindNeighbors’ and ‘FindClusters’ functions were used for identifying actual cell clusters. Unbiased cell type recognition was visualised using uniform manifold approximation and projection (UMAP). Cell types were manually annotated based on classical markers, referring to previous studies [36, 37, 38] and the CellMarker database (http://xteam.xbio.top/CellMarker). Of note, after identifying epithelial cells based on canonical markers, we further dissected and subclustered cells utilising the Seurat R package, leveraging the integrated principal component analysis (PCA) dimensions (specifically, the first 17 PCs). The resolution parameter was set to 0.8, which yielded the delineation of 17 distinct epithelial cell subclusters. To accurately annotate these subclusters, we examined the expression of known marker genes for different epithelial cell states.

### 2.7 Pseudotime Analysis and Cell–Cell Communication Analysis

Pseudotime trajectories analysis can predict the pathways of cell differentiation, development, and apoptosis, and help understand the transition process between different cell types [39]. The trajectory analysis was conducted utilising the ‘Monocle2’ package to construct a differentiation trajectory of epithelial cells. The ‘DDRTree’ function was applied to reduce the dimensions with default settings. The trajectory was then visualised using the ‘plot_cell_trajectory’ function. The established unusual communication network can reveal the mechanisms of tumour progression, unveil new therapeutic targets, and achieve precise interventions [40]. Cell–cell communication analysis was performed using the R package ‘CellChat’.

### 2.8 Beyondcell for Determining the Sensitivity of Different Cells to Drugs

The Beyondcell algorithm analyzes scRNA‐seq data to identify the response of different cell subpopulations to common chemotherapy agents and determine specific treatments for unique cancers [41]. The algorithm preprocesses the data and then calculates the Beyondcell score (BCS) based on single‐cell expression matrices and drug‐signature collections. The BCS is an indicator of the sensitivity of each cell to the drug. Beyondcell also calculates the switch point, which is a quantitative measure of drug response homogeneity across an entire single‐cell dataset and reflects cell variability. Using the BCS matrix and switch point, we generated a UMAP visualisation to illustrate the drug‐response therapeutic clusters.

### 2.9 Immunohistochemistry (IHC) Staining

Paraffin specimens from 19 patients who underwent PCa surgery were retrospectively obtained from the First Affiliated Hospital of Anhui Medical University, China. The sections were deparaffinised, rehydrated, and subjected to antigen retrieval. A 5% bovine serum albumin block preceded incubation with primary antibodies overnight at 4°C, followed by secondary antibody incubation. Diaminobenzidine was used to visualise immunoreactivity. The staining intensity was recorded as follows: 0, negative; 1, weak positive; 2, moderate positive; and 3, strongly positive. The staining area was indicated as follows: 0, 0%; 1, 1%–25%; 2, 26%–50%; 3, 51%–75%; and 4, > 76%. The ultimate score was defined as the intensity score multiplied by the staining area [42]; primary antibodies used in IHC staining included CENPA (Zenbio, cat. #R10068, China, 1:150) and CKS1B (Affinity Biosciences Ltd., cat. #DF3221, China, 1:200).

### 2.10 Cell Culture and Small Interfering RNA (siRNA) Transfection

The PC3 cell line was cultured in RPMI 1640 medium supplemented with 10% fetal bovine serum and 1% penicillin–streptomycin solution containing 100 U/mL penicillin and 100 mcg/mL streptomycin. The incubation process was carried out at 37°C and under an atmosphere of 5% CO_2_. The PC3 cell line was provided by Procell Life Science & Technology Co. Ltd. (Wuhan, China). For transfection, once the cell density reached 60%–70%, 50 pmol/mL of siRNA was transfected into the cell line using Lipofectamine 3000 (Invitrogen, Carlsbad, CA, USA) for 8 h. Subsequently, the transfection mixture was removed, and the cells were cultured under standard conditions. Functional assays were performed 48 h post‐transfection. The siRNAs targeting CENPA and CKS1B were purchased from JTSBIO Co. Ltd. (Wuhan, China).

### 2.11 Western Blot

Cells were lysed with RIPA buffer (Beyotime) to extract total protein, and the concentration was determined. Equal protein amounts were mixed with loading buffer, denatured at 100°C for 3 min, and separated by SDS‐PAGE (80 V for 30 min, then 120 V for 1–2 h). Proteins were transferred to a membrane at 300 mA for 60 min. The membrane was blocked for 1 h, incubated overnight at 4°C with primary antibodies (CENPA (Zenbio, cat.#R10068, 1:1000) and CKS1B (Affinity Biosciences LTD, cat.#DF3221, 1:1000)), then washed and incubated with an HRP‐conjugated secondary antibody (1:5000, Beijing ComWin Biotech) for 1 h at room temperature. After washing, protein signals were detected using a chemiluminescence imaging system (Bio‐Rad) and analysed.

### 2.12 Cell Proliferation and 5‐Ethynyl‐2′‐Deoxyuridine (EdU) Assays

PC3 cells from the NC group, CENPA knockdown group, and CKS1B knockdown group were seeded in 6‐well plates with 500 cells per well and cultured in standard cell culture medium for 2 weeks until cell colonies were formed. Then, colonies were washed with PBS, fixed with 4% paraformaldehyde, and stained with crystal violet for visualisation. Only colonies consisting of more than 30 cells were selected for further analysis.

In the same way, cells from the three groups (500 cells/well) described above were seeded into 96‐well plates. After cell attachment (after 8 h), carefully aspirate the original medium. Subsequently, a mixture of 100 μL of 10% CCK‐8 reagent (Beyotime) and complete medium is added to each well. The plate is then incubated in a 37°C incubator protected from light for 2 h. After incubation, the optical density (OD) value at 450 nm was measured and recorded using a microplate reader. This measurement process is repeated every 24 h for a total of 6 days, and all operations are carried out in strict accordance with the manufacturer's instructions (Beyotime, C0071S).

To evaluate cell proliferation, DNA synthesis was tagged using 5‐ethynyl‐2′‐deoxyuridine (EdU). PC3 cells were arrayed into a 96‐well plate at a density of 3000 cells per well, and then nurtured within a humidified incubator maintained at a physiological temperature of 37°C, under an atmosphere enriched with 5% CO_2_. The experiment was conducted using the BeyoClick EdU‐555 Cell Proliferation Detection Kit. After EdU labeling, the culture medium was carefully aspirated, and the cells were fixed with 4% paraformaldehyde at room temperature for 15 min. Following fixation, the cells were permeabilised with PBS containing 0.3% Triton X‐100. After a washing procedure, a click reaction solution was added to each well and incubated at room temperature in the dark for 30 min. The cell nuclei were stained with Hoechst dye. Finally, fluorescence imaging was carried out using a confocal high‐content imaging system to discern the accurate condition of cell proliferation.

Images were captured at wavelengths of 460 and 500 nm using an inverted fluorescence microscope. Finally, the obtained images were processed and analysed using ImageJ software.

### 2.13 Statistical Analysis

All the statistical analyses were carried out using R (version 4.3.0). Continuous data were analysed using an independent *t*‐test or the Wilcoxon signed‐rank test. The Fisher accuracy test was used to analyse the classified data. *p* < 0.05 was considered to indicate statistical significance.

## 3 Results

### 3.1 Construction and Evaluation of the PCMP Signature

**FIGURE 1:**
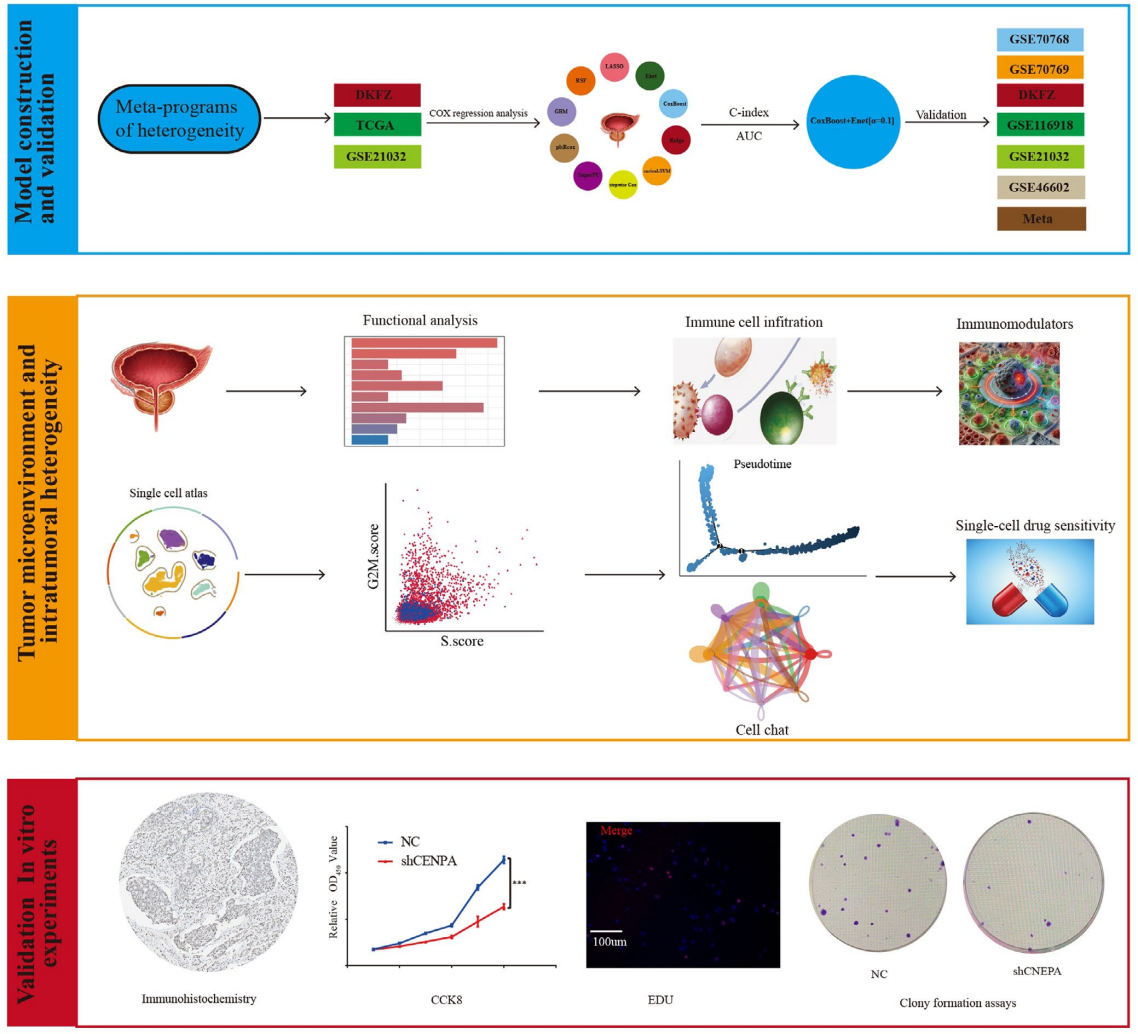
Study workflow schematic.

**FIGURE 2:**
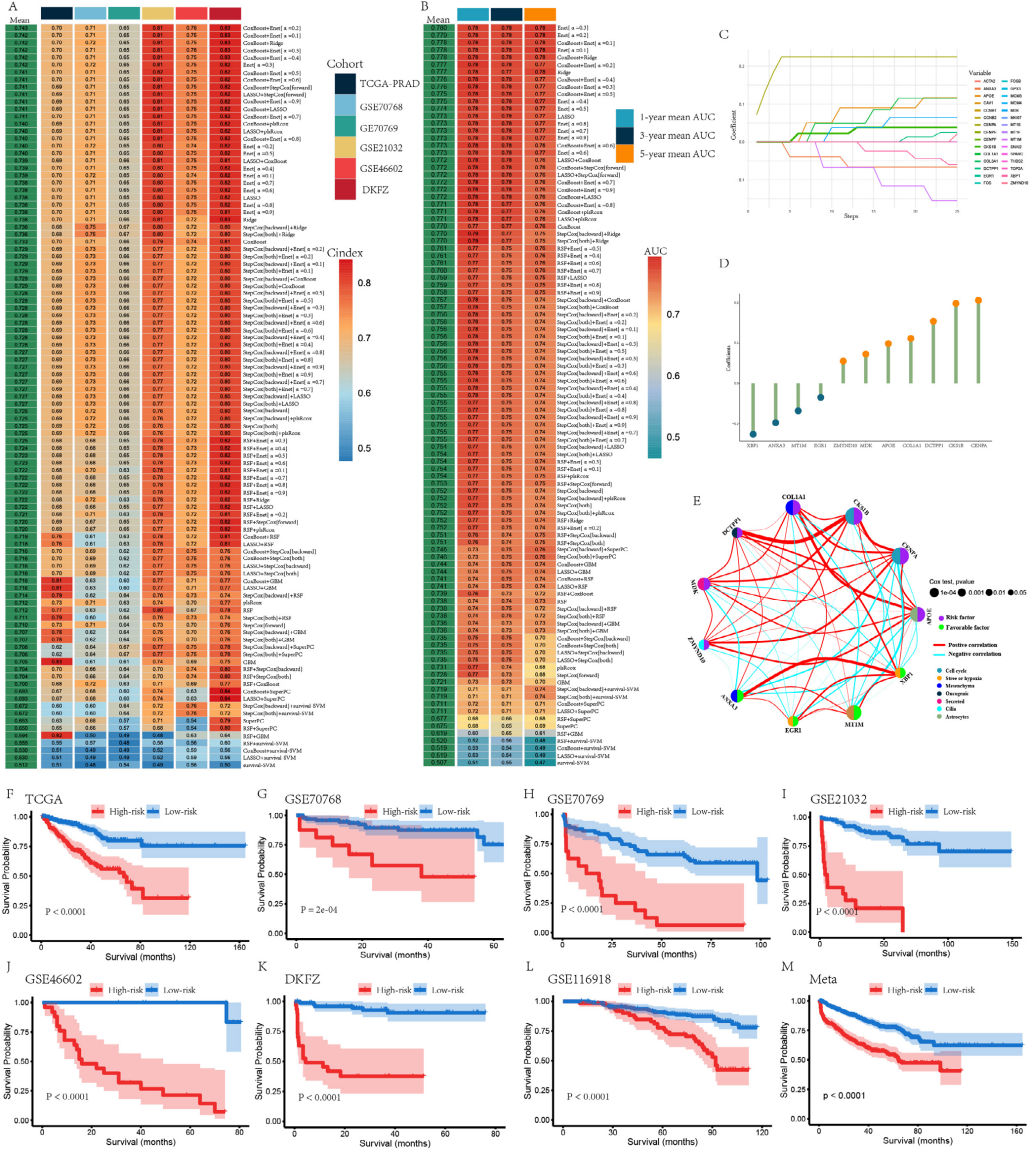
Construction and validation of the prostate cancer meta‐program (PCMP) signature utilising 101 combinations of 10 algorithms. (A) C‐index and (B) 1‐, 3‐ and 5‐year average AUC values of 101 types of prediction models were calculated based on a 10‐fold cross‐validation framework in six cohorts. (C) Hub genes were selected through the application of the CoxBoost algorithm. (D) Coefficients of the PCMP signature were obtained by conducting Enet (*α* = 0.1) algorithm. (E) Gene interaction network diagram. (F–M) Kaplan–Meier (KM) curves of relapse‐free survival were conducted according to the PCMP across TCGA‐PRAD, GSE70768, GSE70769, GSE21032, GSE46602, DKFZ, GSE116918 and Meta cohorts.

Figure 1 illustrates the analytical process workflow. Based on ITH meta‐programs obtained from a previous study [25], we retained 398 ITH‐related genes for subsequent analysis after eliminating any redundant entries. To refine the filtering process and identify factors with profound prognostic significance in the majority of patients with PCa, we rigorously applied univariate Cox regression across the TCGA, DKFZ and GSE21032 datasets, resulting in a final screening of 31 genes (Figure S1). Moreover, to avoid overfitting and enhance the generalisability of our model, we employed several machine learning approaches, including Enet, Ridge, LASSO, CoxBoost, GBM, RSF, StepCox, plsRcox, survival‐SVM and SuperPC, for variant filtering. We used these approaches and their 101 combinations to establish a consensus model. For each model, we calculated the C‐index and the average AUC for 1‐, 3‐ and 5‐year survival predictions. These metrics were visualised in two heatmaps (Figure 2A,B).

From consolidating the results above, CoxBoost and Enet (*α* = 0.1) were defined as the most optimal combination with a mean C‐index and AUC of 0.742 and 0.778, respectively. An 11‐gene rational signature encompassing ANXA3, APOE, CENPA, CKS1B, COL1A1, DCTPP1, EGR1, MDK, MT1M, XBP1 and ZMYND10 was detected using these approaches, and the correlated coefficients were −0.09782575, 0.09870858, 0.20617906, 0.19841336, 0.11160449, 0.15425631, −0.03496635, 0.07269574, −0.06821611, −0.12656854 and 0.05515923, respectively (Figure 2C,D). A correlation network illustrated the potential interactions among the model genes and their influence on PCa, while elucidating the classification of various biological functions (Figure 2E). Furthermore, constructed KM curves illustrated a stark discrepancy in survival outcomes between the risk subgroups (*p* < 0.001), delineated by the best cut‐off value across all datasets (Figure 2F–M).

### 3.2 Evaluation of the PCMP Signature Performance

**FIGURE 3:**
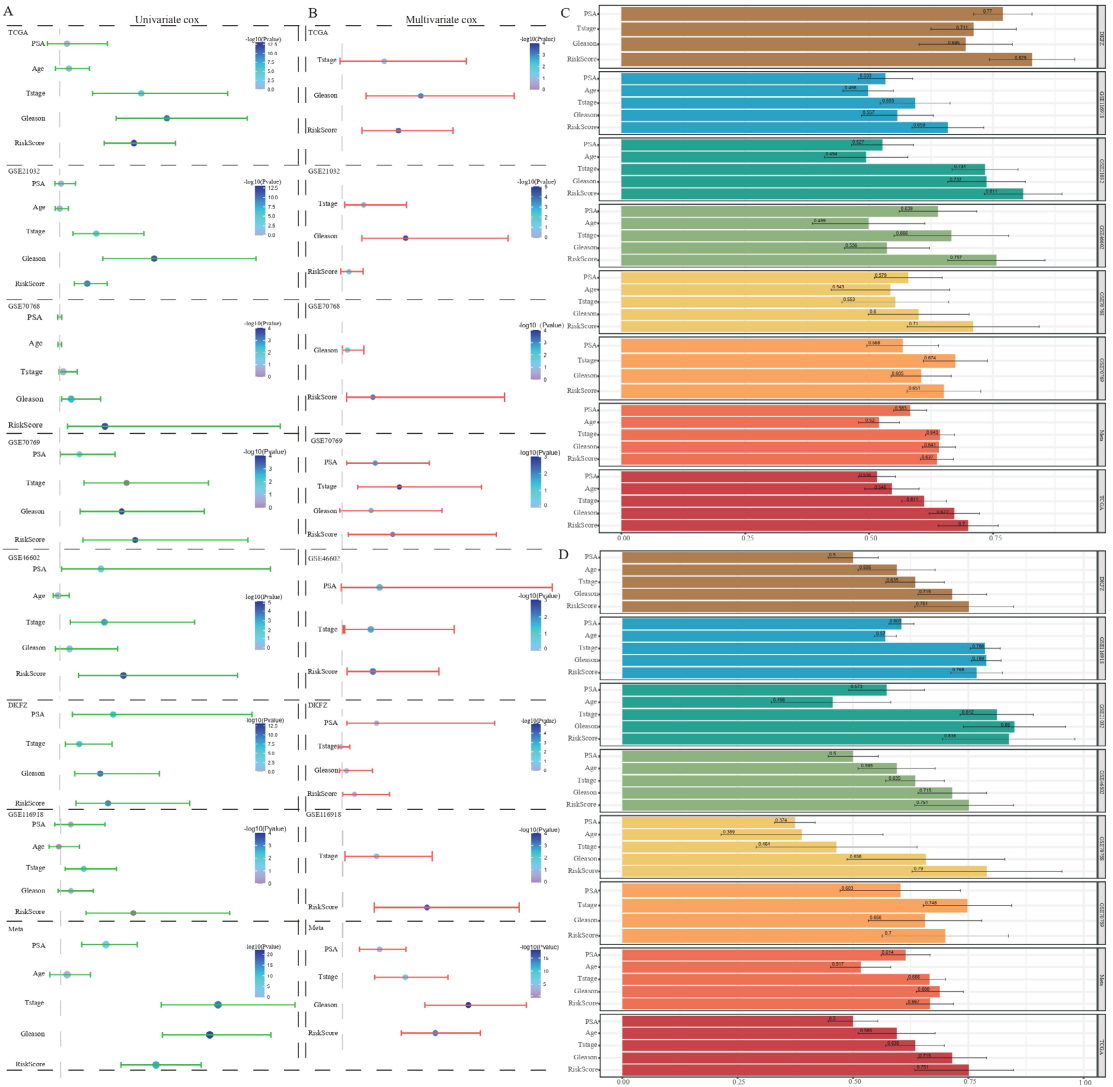
Evaluation of PCMP signature performance in external cohorts. The results of (A) Univariate and (B) multivariable Cox regression analysis of PCMP signature and clinicopathological parameters across TCGA‐PRAD, GSE70768, GSE70769, GSE21032, GSE46602, DKFZ and GSE116918 datasets are depicted by forest plots. (C) C‐index and (D) AUC values of PCMP signature and clinicopathological indicators exhibited by bar plots in eight cohorts.

We performed univariate and multivariate Cox regression analyses to ascertain the robustness and generality of our model. The forest plots (Figure 3A,B) clearly demonstrated that the PCMP signature possessed independent prognostic significance across all datasets examined, except for the GSE21032 dataset, a discrepancy that may be attributed to biases inherent in geographical limitations or statistical fluctuations. Additionally, we compared the C‐index and AUC between the risk score and other clinical indicators to corroborate model performance (Figure 3C,D). The calculated C‐index of risk scores across the DKFZ, GSE116918, GSE21032, GSE46602, GSE70768, GSE70769, meta‐, and TCGA datasets was identified to be 0.829, 0.659, 0.811, 0.757, 0.71, 0.651, 0.637 and 0.700, respectively. Consistently, the AUC values of 0.751, 0.768, 0.838, 0.751, 0.790, 0.700, 0.667 and 0.751 for the risk score across all cohorts also indicated a high level of predictive performance.

### 3.3 Functional Enrichment Analysis and Immune Infiltration

**FIGURE 4:**
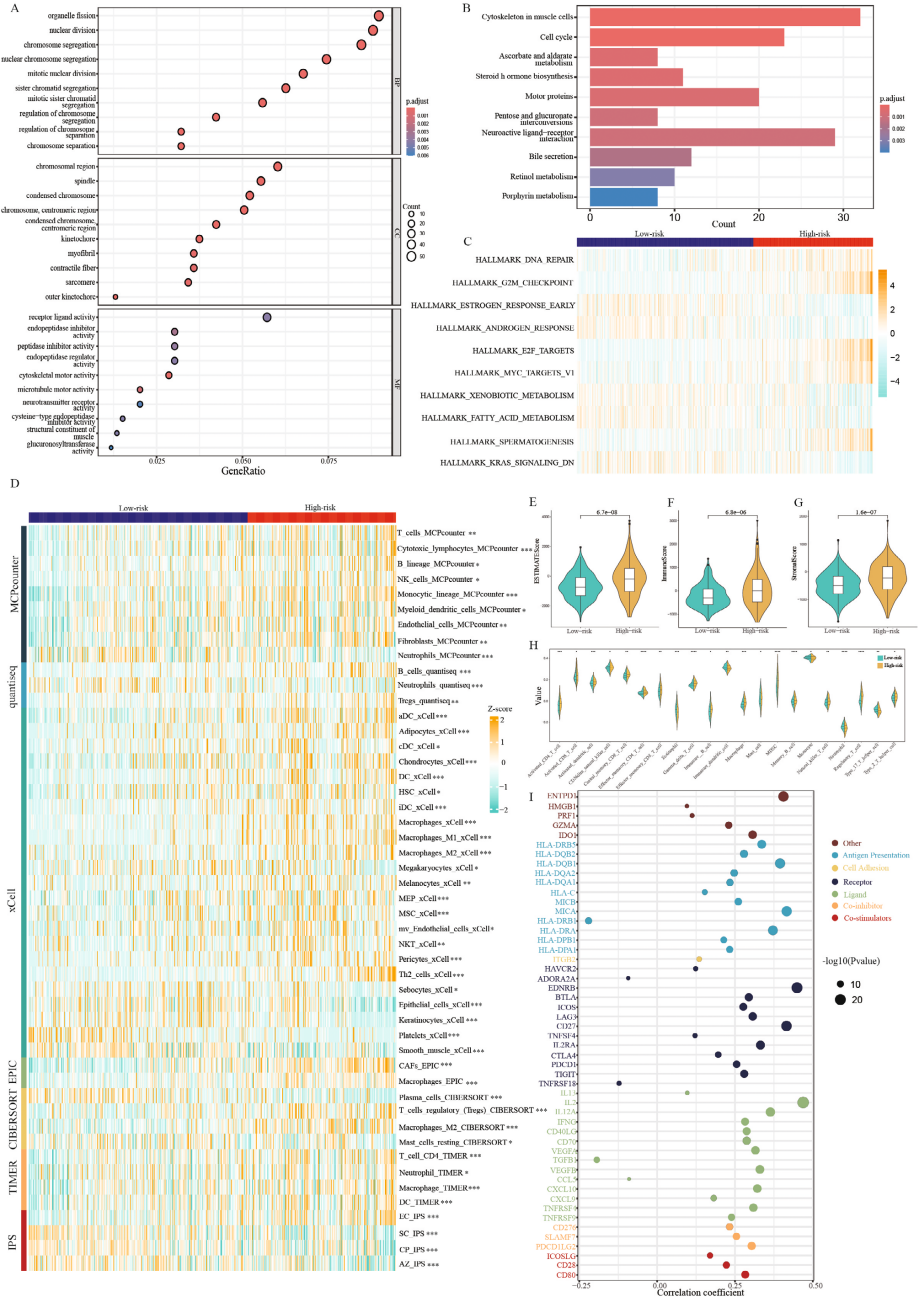
Functional enrichment analysis and immune infiltration. (A) GO and (B) KEGG enrichment analysis. (C) A heatmap displaying the results of hallmark enrichment analysis. (D) Differential abundance of immune cells via various algorithms in the high‐ and low‐risk subgroups depicted by heatmaps. (E–G) Comparisons of the differences between two risk subgroups in terms of estimate, immune, and stromal scores. (H) A half‐sided violin diagram of the abundance of 28 immune cells in the high‐ and low‐risk subgroups. (I) Correlation coefficients between risk score and immunomodulators. *p < 0.05, **p < 0.01, ***p < 0.001.

To further determine the potential biological functions of the prognostic indicators, we performed GO enrichment analysis, the results of which were presented as dot plots (Figure 4A). In terms of biological processes, the model genes played pivotal roles in nuclear and chromosomal separation. Similarly, they were strongly correlated with chromosomal regions and condensed chromosomes. Furthermore, the activities of receptor ligands, endopeptidase inhibitors, peptidase inhibitors and endopeptidase regulators were potentially regulated by prognostic genes. KEGG enrichment analysis about the PCMP signature revealed that it predominately had implications for muscle cell cytoskeletons, cell cycle, neuroactive ligand‐receptor interaction, motor proteins and multiple metabolic processes (Figure 4B). Additionally, the GSEA results depicted in a heatmap demonstrated the underlying molecular pathways (Figure 4C). In the high‐risk subgroup, signalling pathways relevant to DNA repair, G2M checkpoint, E2F targets, MYC targets and spermatogenesis were activated. In contrast, oestrogen and androgen responses, xenobiotic and fatty acid metabolism, and KRAS signalling pathways were mainly enriched in the low‐risk subgroup.

Using the various algorithms mentioned above, the abundance of most immune cells, such as T cells, B cells and myeloid cells, was higher in the high‐risk subgroup (Figure 4D). In contrast, parenchymal cells containing keratinocytes, epithelial cells and smooth muscle cells were enriched in the low‐risk subgroup. The TME immune score analysis illustrated that the high‐risk subgroup consistently possessed remarkably higher estimate, immune and stromal scores (*p* < 0.001), corroborating the heatmap results (Figure 4E–G). To confirm the reliability of the previously mentioned algorithms, we evaluated the distribution of 21 immune cells among the different risk subgroups. A half‐sided violin plot (Figure 4H) clarified the presence of general differences in the distribution of immune cells between the two subgroups. In addition, common functional immune cells encompassing CD8^+^ T cells, CD4^+^ T cells, natural killer cells, and macrophages were predominantly centred on high‐risk patients, corresponding to the heatmap results.

To assess the potential correlation between the PCMP and immune checkpoints, which could guide the application of immunotherapy, we explored the correlation between the risk score and multiple functional immune checkpoints (Figure 4I). As indicated by the correlation coefficients, the checkpoints related to antigen presentation, cell adhesion, co‐inhibitory and co‐stimulatory signals and ligand‐receptor interactions exhibited a positive correlation with the risk score. This suggests a robust association between these immune regulatory mechanisms and the risk score.

### 3.4 Establishing a Single‐Cell Atlas for PCa

**FIGURE 5:**
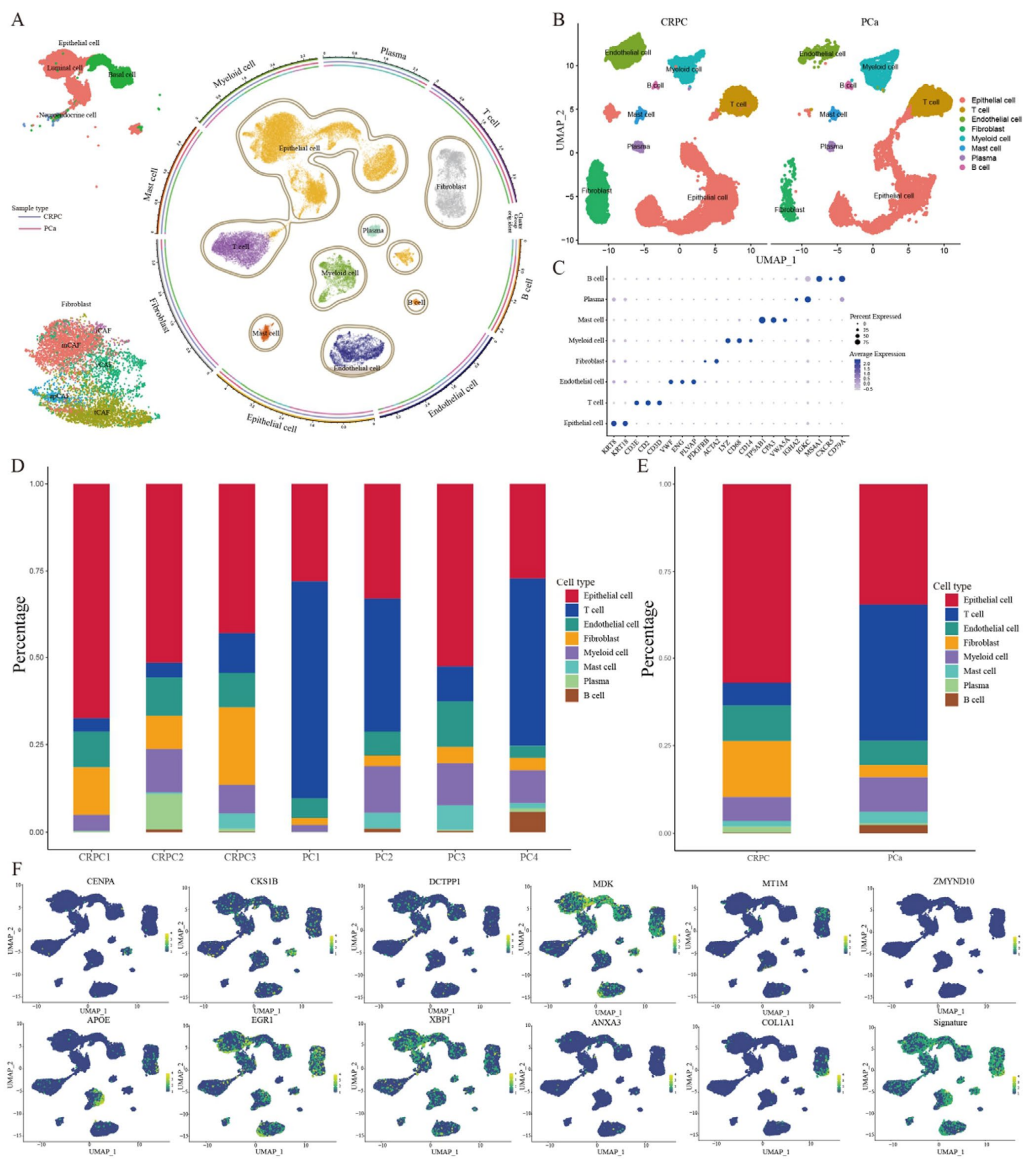
A single‐cell atlas constructed from seven patients. (A) Uniform manifold approximation and projection (UMAP) plots of 47,156 high‐quality cells. (B) UMAP plot coloured by cell types showing eight major cell types in PCa and CRPC. (C) Dot plot showing the markers of each cell type. (D) Bar plot showing the cell proportion in each sample. (E) Average proportion of cell types in different groups. (F) UMAP plot indicating the enrichment of model genes, containing ANXA3, APOE, CENPA, CKS1B, COL1A1, DCTPP1, EGR1, MDK, MT1M, XBP1 and ZMYND10 in PCa tissues.

To reveal the role of prognostic genes in PCa, we collected three CRPC and four PCa samples to construct a TME‐related single‐cell atlas. After stringent quality control and exclusion of low‐quality cells, cells with similar expression patterns were divided into the same cluster, and eight cell types were generated for PCa and CRPC, namely epithelial cells, T cells, endothelial cells, fibroblasts, myeloid cells, mast cells, plasma cells and B cells (Figure 5A,B). Cell clusters were rationally annotated, and the expression of marker genes in the eight cell types was presented as a dot plot (Figure 5C). Bar diagrams (Figure 5D,E) revealed that CRPC was characterised by a remarkable increase in the proportion of epithelial and fibroblast populations. Conversely, the levels of T lymphocytes and myeloid cells were notably reduced in CRPC. Furthermore, we explored the distribution of model genes in different cell types and found that they were predominantly enriched in epithelial cells and fibroblasts (Figure 5F). In summary, it is reasonable to speculate that PCMP may be crucial in tumour development and progression by inducing the malignant transformation of epithelial cells in CRPC.

### 3.5 Functional and Pseudotime Analysis of Epithelial Cells in PCa

**FIGURE 6:**
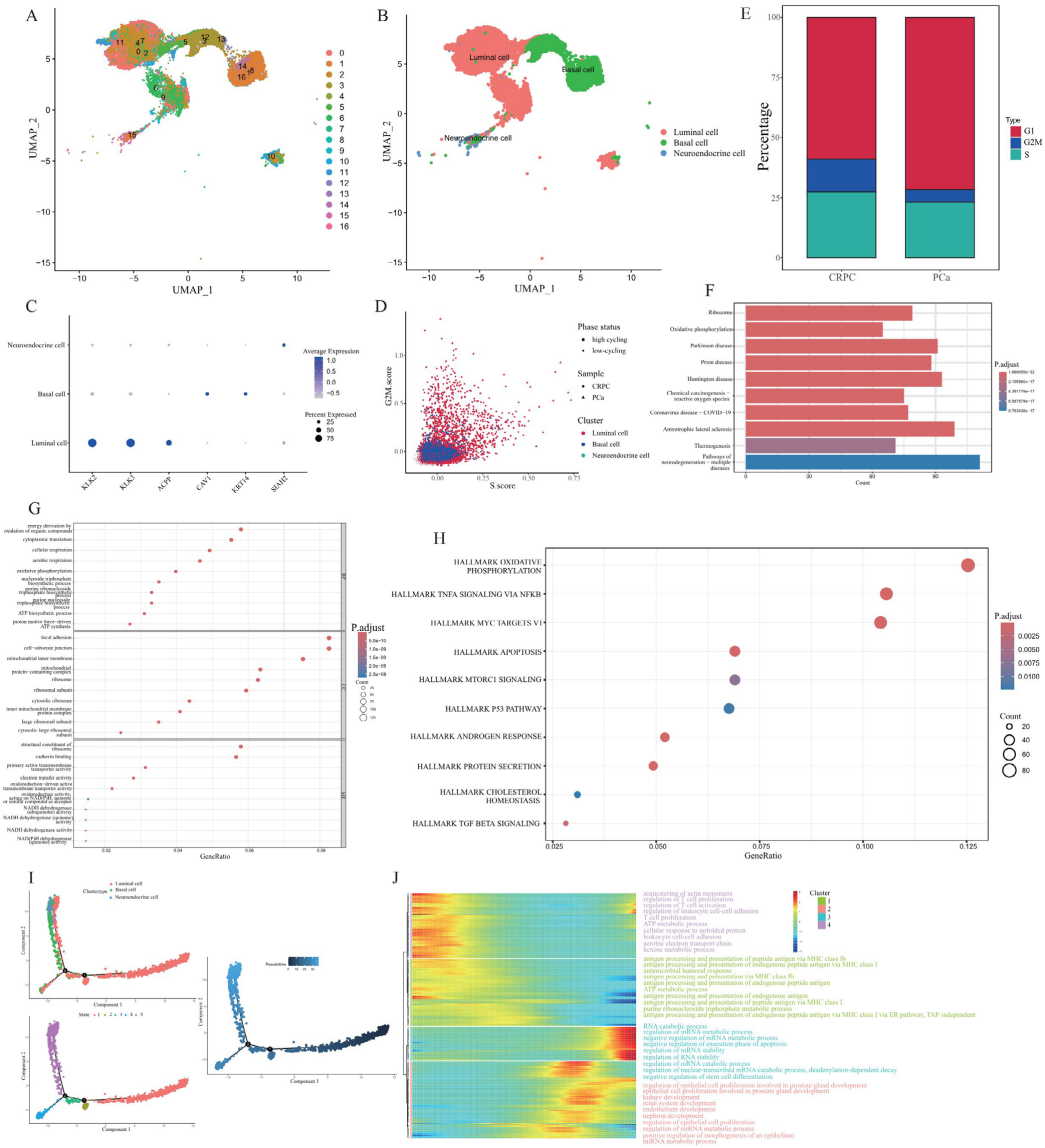
Identification and functional characterisation of epithelial cells. (A) UMAP visualisation delineates 17 distinct cellular clusters within two datasets comprising 24,317 cells. (B) Epithelial cells separated into three cell types including luminal cells, basal cells, and neuroendocrine cells. (C) A dot plot showing the expression profiles of marker genes for each cell type. (D) Cell cycle status distribution of different samples. Different shapes indicate different samples and different colours indicate different cell types. (E) A bar plot delineating the cell cycle status in different samples. (F) KEGG, (G) GO and (H) hallmark enrichment analyses of hub genes expressed by luminal cells. (I) Monocle 2 trajectory analysis of epithelial cells annotated by cell subgroups. (J) A heatmap displaying the alterations of signalling pathways aligned with pseudotime.

Because the model genes were predominantly located within the epithelial compartment, we subsequently delineated the cell population into 17 sub‐clusters for a more nuanced analysis (Figure 6A). With reference to conventional cell markers, the entire cluster was annotated as luminal, basal, or neuroendocrine cells (Figure 6B). The marker genes' expression profiles of these sub‐clusters were visually represented in a dot plot (Figure 6C). Alternatively, we calculated the cell cycle‐related score and found that luminal cells, especially those originating from CRPC, exhibited high cycling characteristics (Figure 6D). The precise distribution of CRPC cells across distinct cell cycle phases, including the gap2, mitosis and synthesis stages, was displayed in a bar chart (Figure 6E). Since luminal cells are crucial for the renewal of tumour cells, we extracted pivotal genes expressed by luminal cells and explored their relevant biological functions. KEGG analysis demonstrated that these play a key role in ribosome metabolism and oxidative phosphorylation (Figure 6F). In terms of biological processes, luminal cells are predominantly engaged in energy derivation by the oxidation of organic compounds, cytoplasmic translation and cellular and aerobic respiration. They constitute a variety of cellular components encompassing focal adhesions, cell‐substrate junctions, mitochondrial inner membranes, mitochondrial protein‐containing complexes and ribosomes. Consistent with this, molecular functions that involve the structural constituents of the ribosome, cadherin binding, primary active transmembrane transporter activity, electron transfer activity and oxidoreduction‐driven active transmembrane transporter activity also require the participation of these genes (Figure 6G). Furthermore, hallmark pathways analysis illustrated that the oxidative phosphorylation‐related signalling, TNFA signalling, MTORC1 signalling and apoptosis‐related signalling pathways were activated prominently in epithelial cells (Figure 6H).

To reveal the optimal developmental trajectory of epithelial cells, we performed a pseudotime analysis of all sub‐clusters (Figure 6I). As shown in Figure 6I, all cells were divided into five different states according to the cell evolution conditions. Based on the colour shades of the pseudotime plot, we determined that the cells differentiated from right to left. This is due to the fact that the darker the colour, the earlier the cells appeared. We hypothesised that epithelial cells transform from luminal to basal cells, and then to neuroendocrine cells. As mentioned earlier, the epithelial clusters were further separated into four clusters. Alterations correlated with biological signalling pathways across communities during evolution, as shown in a heatmap (Figure 6J). In the first cluster, signalling pathways related to T cell activation and proliferation were significantly downregulated. Cell metabolism‐related pathways, including the ATP metabolic process, aerobic electron transport chain and hexose metabolic process, were also downregulated. In cluster 2, antigen processing and presentation of endogenous peptide antigens via major histocompatibility complex (MHC) class I, which serves as the predominant biological process, were remarkably suppressed. Conversely, the regulation process of mRNA metabolic process and stability was highly activated in both clusters 3 and 4, and signalling pathways correlated to epithelial cell proliferation, which contribute to the development of the kidney and prostate, were also enriched.

### 3.6 Cell Chat and Drug Sensitivity Analysis

**FIGURE 7:**
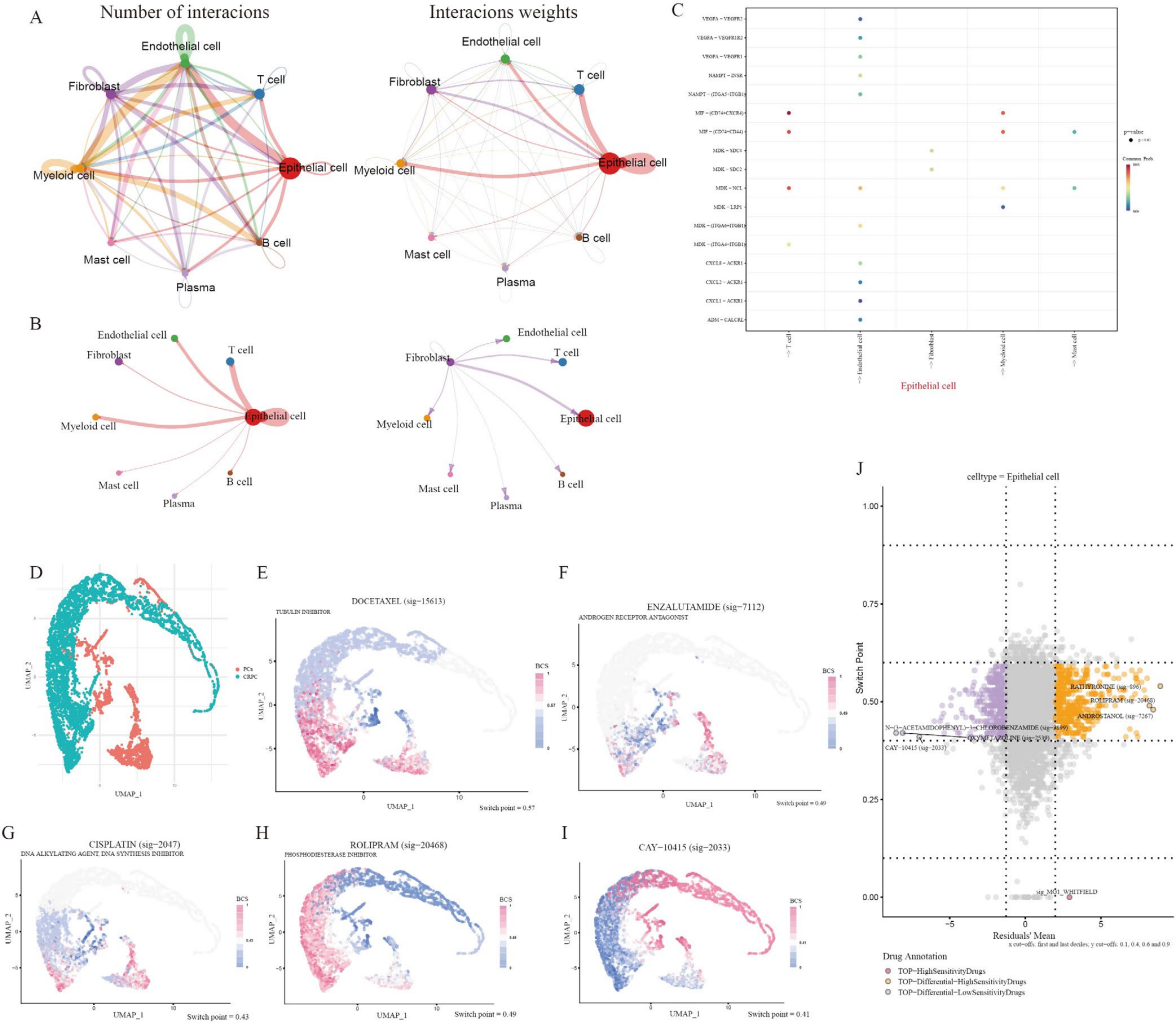
Cell–cell communications and single cell drug sensitivity analysis. (A) Network diagrams reflecting the numbers and weights of inter‐cellular communication. (B) Circle plots showing the interaction intensities of the outgoing interactions of epithelial cells (left) and fibroblast (right). (C) A heatmap showing the enriched ligand‐receptor and signalling pathways interaction intensities of epithelial cells with other cell types. (D) Beyondcell analysis calculating the BCS of each cell in the UMAP dimension. (E–I) BCS score distributions of docetaxel, enzalutamide, cisplatin, rolipram, and cay‐10,415 in the UMAP dimension. (J) A bc4Squares plot describing the detailed positions of 5201 drugs when comparing endothelial cells with other cells.

We constructed an interaction network between different cell types; the thickness and number of connecting lines reflected the reciprocal weights and numbers between cell types (Figure 7A). As the model genes were predominantly distributed in epithelial cells and fibroblasts, we explored the potential correlation between epithelial cells or fibroblasts and other cells. The results demonstrated that epithelial and T cells were the most closely linked (Figure 7B). The macrophage migration inhibitory factor (MIF) signal was obviously activated when epithelial cells interacted with T and myeloid cells. Simultaneously, signal transduction is principally impacted via the MIF‐(CD74 + CXCR4) and MIF‐(CD74 + CD44) ligand‐receptor pairs (Figure 7C).

The drug sensitivity of individual cancer cells impacts the development of rational therapeutic programmes. By calculating the switch points, we measured the sensitivity of various parts of PCa and CRPC to common anti‐tumour drugs, including docetaxel, enzalutamide, and cisplatin, at the single‐cell level (Figure 7D–I). It is noteworthy that distinct cells within the tumour exhibit varied responses to chemotherapeutic agents because of cell heterogeneity. This variability may be a critical factor in the development of drug resistance in patients with PCa. Notably, the observation that rolipram and CAY‐10415 have complementary sensitivities to PCa and CRPC treatment can provide new insights into individualised therapy. The overall responsiveness of the epithelial cells to multiple agents was presented in a volcano plot (Figure 7J). Rathyronine, rolipram and androstanol were highly sensitive agents for PCa and CRPC. Conversely, N‐(3‐acetamidophenyl)‐3‐chlorobenzamide, oxymetazoline and CAY‐10415, when applied to patients, may result in poorer outcomes.

### 3.7 IHC Validation of CENPA and CKS1B Expressions

**FIGURE 8:**
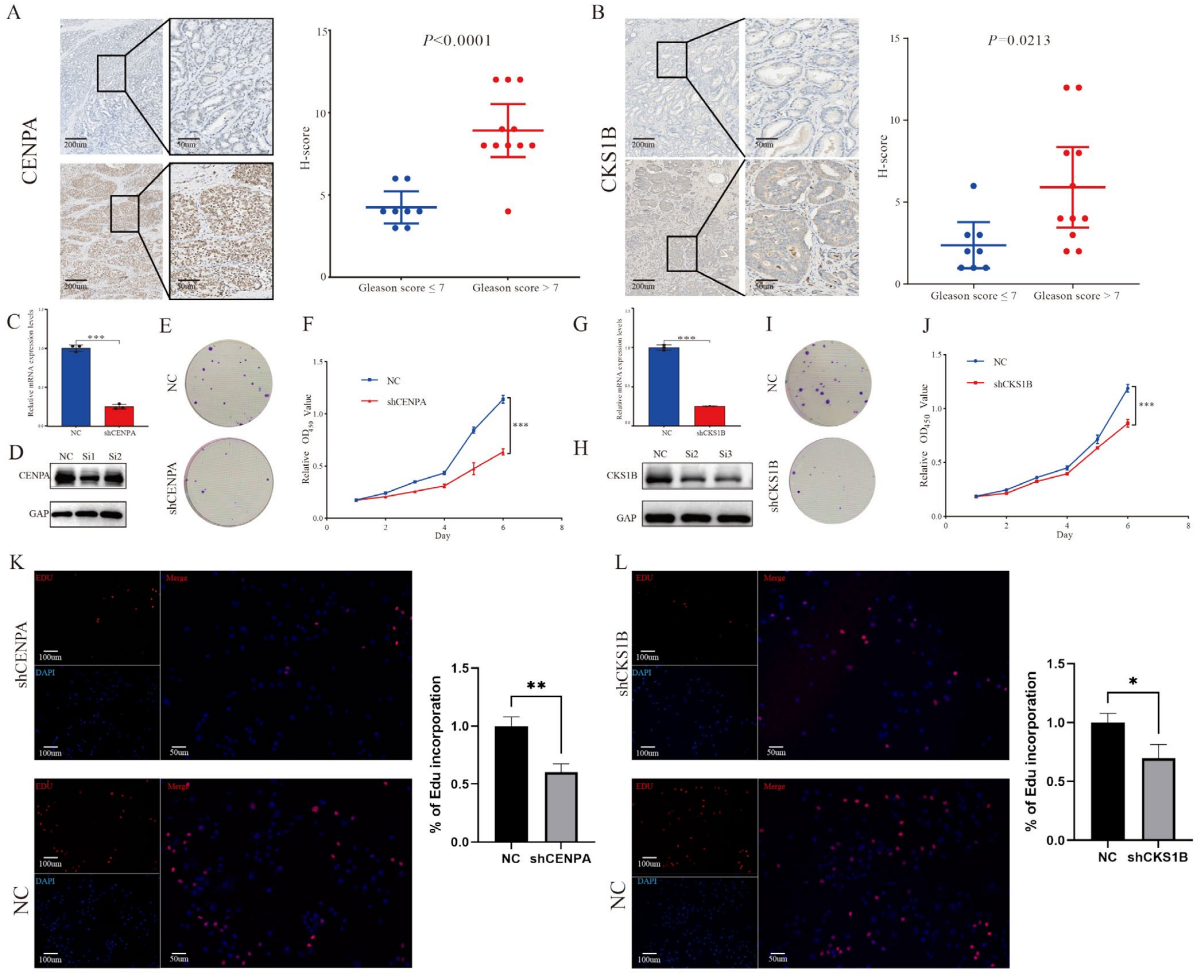
CENPA and CKS1B are overexpressed in PCa and promotes PCa cell proliferation. (A, B) Representative pictures showing the different protein levels of CENPA and CKS1B in patients with PCa with different Gleason scores. (C) siRNAs inducing gene silencing, reducing CENPA mRNA and protein levels in PC3. (D) Western blot analysis shows successful knockdown of CENPA protein. (E, F) CENPA silencing clearly inhibits the colony formation and proliferation of PC3 cells. (G) siRNAs inducing gene silencing, reducing CKS1B mRNA and protein levels in PC3. (H) Western blot analysis shows successful knockdown of CKS1B protein. (I, J) CKS1B silencing clearly inhibits the colony formation and proliferation of PC3 cells. (K) EdU staining assay elucidated remarkably fewer EdU‐positive cells in shCENPA group compared with NC group. (L) EdU staining assay elucidated remarkably fewer EdU‐positive cells in shCKS1B group compared with NC group. *p < 0.05, **p < 0.01, ***p < 0.001.

CENPA and CKS1B were selected for subsequent validation because of their high weights in the risk‐score formula. The standard definition of the protein level is elaborated in the methods section, and the expression of CENPA and CKS1B was significantly upregulated in malignant tissues (Gleason score ≤ 7 vs. Gleason score > 7, all *p* < 0.05) (Figure 8A,B).

### 3.8 CENPA and CKS1B Silencing Affect Malignant Phenotype

To further investigate the actual functions of CENPA and CKS1B, we constructed siRNAs that silenced their target genes. Similarly, the results of quantitative polymerase chain reaction (qPCR), alongside Western Blot (WB) ascertained the efficacy of siRNAs targeting CKS1B and CENPA in silencing results (Figure 8C,D,G,H). Since the superior transformed effect of CENPA‐Si1 and CKS1B‐Si2 was observed, they were chosen for subsequent analysis. After suppressing CENPA and CKS1B expression, colony formation and CCK8 assays demonstrated that cell viability decreased significantly compared to that in the NC group (all *p* < 0.05) (Figure 7E,F,I,J). An EdU assay was employed to evaluate proliferation activation, showing that the proliferative capacity was inhibited after knocking down genes (Figure 7K,L).

## 4 Discussion

The inherent genomic and phenotypic heterogeneity exhibited in PCa contributes to suboptimal therapeutic outcomes in advanced stages. Consequently, exploring novel diagnostic and therapeutic markers is critical for risk stratification and understanding the fundamental mechanisms driving PCa progression [43, 44, 45, 46, 47]. By utilising an ITH atlas constructed through scRNA‐seq, we dissected meta‐programs undergoing synergistic mutations in various cancers or different cells originating from the same cancer species [25]. Meta‐programs were confirmed to characterise the cellular state, inducing the malignant transformation of cells, and were primarily enriched in biological processes such as the cell cycle, stress or hypoxia, interferon and MHC II. Mutations in cell cycle‐related indicators, caused by somatic alterations, can further affect the transformation from PCa to CRPC [48, 49, 50]. A systematic review and meta‐analysis also implied that the cell cycle score may be a nascent index for guiding the comprehensive therapy of patients with PCa [51]. Exploring the relationship between meta‐programs and the cell cycle can provide novel insights into the growth, progression, and treatment resistance of PCa cells.

Underlying the 10‐fold cross‐validation framework, 101 combinations of 10 algorithms were applied to the training and validation cohorts to construct a robust PCMP signature containing ANXA3, APOE, CENPA, CKS1B, COL1A1, DCTPP1, EGR1, MDK, MT1M, XBP1 and ZMYND10. Through KM analysis, as well as univariate and multivariate Cox regression analyses, the robustness and superiority of the PCMP signature were demonstrated, illustrating that it was a strong predictor of clinical outcomes.

Based on the results of the GO enrichment analysis, biological processes correlated with cell proliferation, such as mitotic nuclear division, chromosome segregation, and sister chromatid segregation, play a major role in the formation and progression of heterogeneity. Regarding evolutionary theory, genomic instability, which contributes to heterogeneity, induces dysregulation of mitotic division and the generation of aneuploidy to enhance aggressiveness [52, 53]. A hallmark pathway enrichment analysis revealed that patients in the high‐risk subgroup had a lower response to androgen and oestrogen, whereas the E2F, MYC and G2M checkpoints were significantly upregulated. Previous studies have shown that E2F family members are positively correlated with the Gleason score, advanced tumour stage and metastasis, and were explored as candidates for measuring the level of heterogeneity [54]. Serving as a regulator of the metabolic requirements for PCa, MYC increases the invasiveness and promotes the cell‐cycle progression of PCa by affecting androgen receptor targets [55, 56, 57]. Alterations in the G2/M checkpoint in multiple cancers, including ovarian, bladder and lung cancers, are relevant to the proliferation of cancer cells [58, 59, 60, 61]. Immune infiltration analysis demonstrated that the high‐risk subgroup possessed a high abundance of immune and stromal cells. Positive feedback regulatory mechanisms are generally considered to exist in the TME of high‐risk samples and drive the progression of PCa [62, 63]. In summary, from a transcriptomic perspective, we can infer that PCMP facilitates irregular cell separation and cell proliferation by enhancing the expression of cell‐cycle relevant regulatory proteins. The biological function of unique immune cells can be altered, thereby contributing to the progression of PCa.

Subsequently, to elucidate the in‐depth function of PCMP, we constructed a single‐cell atlas, re‐separated all epithelial cells into 17 clusters, and annotated them as luminal, basal, and neuroendocrine cells for further detection. All epithelial cells originating from PCa and CRPC were evaluated regarding their practical stages in the cell cycle. A boxplot and scatterplot showed that CRPC has superior proliferative activity, and luminal cells have the highest cycling speed. Subsequent results illustrated that marker genes primarily distributed in luminal cells were enriched in oxidative phosphorylation and other energy production‐related pathways. Song et al. traced the evolution of epithelial cells and discovered that luminal cell transformation and loss of basal cells accounted for tumour progression [64, 65]. Previous studies have also demonstrated that oxidative phosphorylation plays a major role as a source of energy in PCa tissues, distinguishing it from other cancer types. This is because zinc is re‐upgraded during malignant transformation, and the tricarboxylic acid cycle is reactivated [66]. Moreover, after defining the developmental directions and distinct status of the three epithelial subtypes, we discovered that biological behaviours, especially antigen processing and presentation, alongside the activation and proliferation of T cells, were remarkably upgraded in luminal cells. Consistent with our discovery, other studies also demonstrated the overactivation of MYC and E2F pathways and their regulated genes, which lead to the remodelling of TME and the regulation of the normal cell cycle [67]. Intriguingly, LXN protein, particularly secreted by luminal cells, has also been proven to exert influence on immune cell differentiation, recruitment and activity [68]. The related process of tumour immune cell evasion by blocking maturation of B and T cells launched by luminal cells has also been revealed. Conversely, the process of mRNA metabolic process, which is confirmed to play a critical role in heterogeneous development [69], was concentrated in basal and neuroendocrine cells. The pseudotime plot illustrated the transformation of biological functions and revealed the differentiation primordial position of malignant luminal cells, consistent with a previous study [70].

By analysing the interactions between various cell types, we determined that the crosstalk between epithelial cells and T cells was the most active. Receptor‐legend networks showed that MIF‐(CD74^+^ CXCR4) and MIF‐(CD74^+^ CD44) were the main modes of interaction among epithelial, T, and myeloid cells. MIF, which is dominatedly expressed in luminal epithelial cells, is overexpressed in PCa and triggers the ERK1/2‐MAPK cascade, thereby inducing cell cycle progression and inhibiting apoptosis by binding with the CD74/CD44‐receptor complex [71, 72]. In conclusion, single‐cell analysis revealed the pivotal role of luminal cells in cancer progression. Through the activation of MIF‐related pathways, luminal cells are in a highly cyclical state, and the extent of oxidative phosphorylation is significantly increased to induce cancer cell growth.

Furthermore, we assessed the sensitivity of different PCa cells to common drugs, including docetaxel, enzalutamide and cisplatin and discovered distinct drug tolerances among them. Rolipram and CAY‐10415, which have complementary therapeutic sensitivities, are potential novel therapeutic drug candidates. Consequently, the responsiveness of distinctive cells to drugs can be determined by single‐cell atlas analysis. Next, we confirmed a strong correlation between the malignant phenotype and two model genes (CENPA and CKS1B) with the highest coefficients. Overexpression of CENPA and CKS1B resulted in proliferative reactivity in PCa. CENPA is essential for metabolic programming, including glutamine metabolism and glycolysis, to induce the progression of endometrial, clonal and triple‐negative cancers [73, 74, 75, 76]. The interaction between CKS1B and PKMYT1 can disrupt the G2/M checkpoint, dysregulate the cell cycle, and promote abnormal proliferation in pancreatic cancer [77, 78].

Our study has some limitations. Because single‐cell and bulk transcriptome data were acquired from public datasets, population bias may exist. Furthermore, in vitro experiments should be conducted to explore in‐depth signalling pathways after identifying potential targets.

## 5 Conclusions

We constructed a PCMP signature to guide PCa therapy. Training and validation based on a combination of machine learning algorithms ensured the robustness of the model in terms of predicting patients' prognosis and responsiveness to common therapies. Notably, prognostic genes were positively correlated with heterogeneity and the cell cycle at the single‐cell level, and we validated their proliferation function at the cellular level. These results can provide novel individualised treatment options for refractory PCa caused by ITH.

## Author Contributions

**Junchao Wu:** conceptualization (equal), data curation (equal), formal analysis (equal), methodology (equal), software (equal), supervision (equal), validation (equal). **Ziqi Chen:** conceptualization (equal), data curation (equal), formal analysis (equal), visualization (equal), writing – original draft (equal). **Wentian Wu:** investigation (equal), methodology (equal), resources (equal), software (equal), supervision (equal), writing – original draft (equal). **Jiaxuan Qin:** conceptualization (equal), data curation (equal), visualization (equal), writing – review and editing (equal). **Rongfang Zhong:** conceptualization (equal), data curation (equal), validation (equal), visualization (equal), writing – review and editing (equal). **Jialin Meng:** conceptualization (equal), data curation (equal), supervision (equal), validation (equal), writing – review and editing (equal). **Yu Yin:** conceptualization (equal), data curation (equal), formal analysis (equal), writing – review and editing (equal). **Peng Guo:** conceptualization (equal), funding acquisition (equal), resources (equal), software (equal), writing – review and editing (equal). **Song Fan:** conceptualization (equal), funding acquisition (supporting), validation (equal), visualization (equal), writing – review and editing (equal).

## Ethics Statement

The approval document has been provided in the appended Supporting Information.

## Consent

The authors have nothing to report.

## Conflicts of Interest

The authors declare no conflicts of interest.

## Supporting information


**Appendix S1:** jcmm70806‐sup‐0001‐AppendixS1.pdf.

## Data Availability

All the data used in this work can be acquired from the Gene‐Expression Omnibus (GEO; https://www.ncbi.nlm.nih.gov/geo/) and the GDC portal (https://portal.gdc.cancer.gov/). Code availability: All the original R codes are available from the corresponding author upon reasonable request.
