# Supplementary material for: Single‐Cell and Bulk RNA Sequencing Highlights Intra‐Tumoral Heterogeneity and Malignant Progression Mechanisms in Prostate Cancer
Source: J Cell Mol Med. 2025 Aug 26;29(16):e70806. doi: 10.1111/jcmm.70806 (PMC12379737; doi:10.1111/jcmm.70806)

Table S1: Summary of the clinicopathological parameters in seven enrolled datasets.

	TCGA-PRAD	GSE70768	GSE70769	GSE21032	GSE46602	DKFZ	GSE116918
Age							
≤60	220	48	-	87	15	105	31
>60	270	63	-	51	21	0	192
T satge							
T1+T2	187	34	46	86	19	68	127
T3+T4	303	77	42	52	17	37	96
Gleason score							
≤7	288	102	74	117	32	91	127
>7	202	9	14	21	4	14	96
Status							
Recurence free	398	92	44	103	14	81	172
Recurred	92	19	44	35	22	24	51

Table S2: Basic quality control statistics and patient samples for the respective combined datasets

Dataset	Sample	Tumor type	Number of cells	Number of genes
GSE193337				
	GSM5793828	PCa	1806	19803
	GSM5793829	PCa	3157	21333
	GSM5793831	PCa	2641	21885
	GSM5793832	PCa	3980	21513
GSE206962				
	GSM6267383	CRPC	18823	21936
	GSM6267384	CRPC	4608	20237
	GSM6267385	CRPC	12141	21114

Fig.S1: Results of univariate Cox regression analysis with prognostic significance in TCGA-PRAD, GSE21032, and DKFZ cohorts

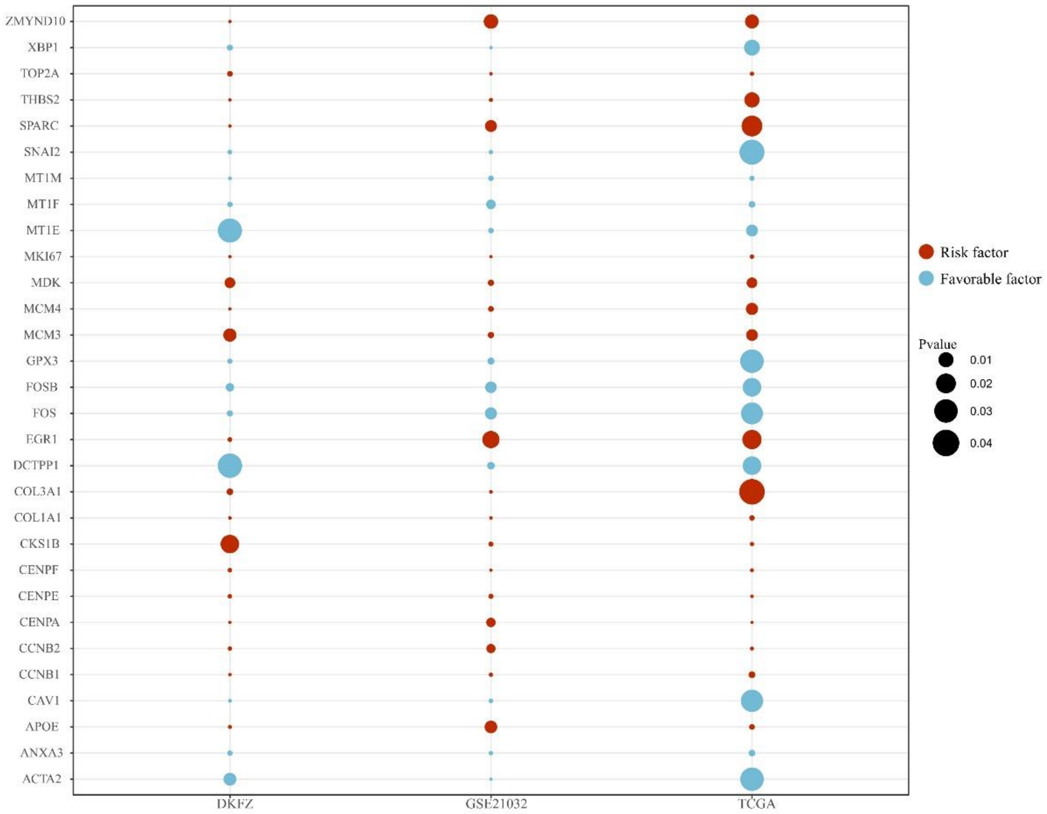

Supplement: Supplementary file 1 — Appendix S1: jcmm70806‐sup‐0001‐AppendixS1.pdf. [file JCMM-29-e70806-s001.pdf]
